# Pseudogene *CSPG4P12* inhibits colorectal cancer progression by attenuating epithelial-mesenchymal transition

**DOI:** 10.1590/1414-431X2024e13645

**Published:** 2024-05-20

**Authors:** Qinqin Song, Hongxue Xu, Hongjiao Wu, Jing Dong, Shanshan Ji, Xuemei Zhang, Zhi Zhang, Wanning Hu

**Affiliations:** 1 Department of Oncology, Hebei Medical University, Shijiazhuang, China; 2 Affiliated Tangshan Gongren Hospital, Hebei Medical University, Tangshan, China; 3 School of Public Health, North China University of Science and Technology, Tangshan, China; 4 Affiliated Tangshan Gongren Hospital, North China University of Science and Technology, Tangshan, China; 5 College of Life Science, North China University of Science and Technology, Tangshan, China

**Keywords:** Colorectal cancer, Pseudogene, CSPG4P12, EMT, Mechanism

## Abstract

Colorectal cancer is one of the most common malignant cancers. Pseudogenes have been identified as oncogenes or tumor suppressor genes in the development of various cancers. However, the function of pseudogene *CSPG4P12* in colorectal cancer remains unclear. Therefore, the aim of this study was to investigate the potential role of *CSPG4P12* in colorectal cancer and explore the possible underlying mechanism. The difference of *CSPG4P12* expression between colorectal cancer tissues and adjacent normal tissues was analyzed using the online Gene Expression Profiling Interactive Analysis 2 (GEPIA2) database. Cell viability and colony formation assays were conducted to evaluate cell viability. Transwell and wound healing assays were performed to assess cell migration and invasion capacities. Western blot was used to measure the expression levels of epithelial-mesenchymal transition-related proteins. Colorectal cancer tissues had lower *CSPG4P12* expression than adjacent normal tissues. The overexpression of *CSPG4P12* inhibited cell proliferation, invasion, and migration in colorectal cancer cells. Overexpressed *CSPG4P12* promoted the expression of E-cadherin, whereas it inhibited the expression of vimentin, N-cadherin, and MMP9. These findings suggested that *CSPG4P12* inhibits colorectal cancer development and may serve as a new potential target for colorectal cancer.

## Introduction

Colorectal cancer (CRC) is the third most common cancer and the second leading cause of cancer-related deaths worldwide with an estimated number of 1.88 million new cases and about 915,880 deaths worldwide in 2020 (1). The initiation, progression, and metastasis of CRC is a multi-step process caused by the accumulation of genetic and epigenetic alterations (2). In recent years, studies revealed that around 16-17% CRC patients carried pathogenic germline variants (3,4). Epigenetic modifications, including DNA methylation, histone modifications, long noncoding RNAs (lncRNAs) and microRNAs (miRNAs), play an important role in the development of CRC (2). During the last decade, due to earlier diagnosis through screening, advances in surgical techniques, and novel targeted therapies, the 5-year relative survival rate for CRC has greatly improved from 50 to 65% (5). However, stage IV CRC patients still have a poor prognosis with 11-15% 5-year survival rate (5). Therefore, there is an urgent need to explore new therapeutic targets of CRC.

Pseudogenes usually refer to a DNA segment structurally similar to a gene but lacking coding function. Recently, increasing evidence indicates that pseudogenes are involved in gene regulation and play important roles in the process of tumor progression (6-8). For example, *RP9P* promotes colorectal cancer progression by regulating the miR-133a-3p/FOXQ1 axis (9). *FLT1P1* inhibits both VEGFR1 and non-cognate VEGF-A expression, suppressing tumor cell proliferation and xenograft tumor growth (10).

Pseudogenes share high sequence homology with their parental genes (11). Pseudogenes and coding genes can talk with each other by competing for the same microRNAs, acting as competing endogenous RNAs (ceRNAs) (11). For example, *PTENP1* regulates its parent gene *PTEN* through the ceRNA mechanism (8). Chondroitin sulfate proteoglycan 4 (encoded by *CSPG4*), a cell surface proteoglycan, plays multiple roles in tumor growth and metastasis (12,13). *CSPG4P12*, as a pseudogene of CSPG4, has been reported to inhibit non-small cell lung cancer (NSCLC) development and tumorigenesis by activating the p53/Bcl2/Bax mitochondrial apoptotic pathway in our previous study (14).

In the present study, we evaluated whether pseudogene *CSPG4P12* regulated the proliferation and metastasis capability of CRC and sought to further explore its potential molecular mechanisms. Our findings may provide a novel insight into the treatment of CRC.

## Material and Methods

### Differential expression analysis

The Gene Expression Profiling Interactive Analysis 2 (GEPIA2) database (http://gepia2.cancer-pku.cn/#analysis), a newly developed interactive web server for analyzing RNA sequencing expression data, was used to evaluate the differential expression of *CSPG4P12* between CRC cancer tissues and adjacent normal tissues. A |log2 fold change (FC)| >1 and P-value <0.05 were set as the cut-off criteria. After entering the gene symbols into the “Gene” column, the expression box plots were automatically generated on the webpage.

### Cell culture and plasmid transfection

Human colorectal cancer cell lines (LOVO, Caco-2, and HCT116) and the human normal colonic epithelial cell line (NCM460) were obtained from American Type Culture Collection (ATCC) (USA). Cells were maintained in RPMI-1640 (EallBio Life Sciences, China) supplemented with 10% fetal bovine serum (FBS) (EallBio Life Sciences) and 1% penicillin and streptomycin (P/S) (EallBio Life Sciences) at 37°C in an incubator with 5% CO_2_.

Cells were seeded onto six-well plates with a density of 1×10^6^ cells/well and transfected at ∼80% confluence with 1 µg *CSPG4P12-pUC57* or control plasmid *pUC57* using Neofect DNA transfection reagent (Neofect Biotech Co., Ltd., China) according to the manufacturer's instructions. After 24 h, the transfected cells were collected for subsequent experiments. The *CSPG4P12-pUC57* plasmid was constructed by Changzhou Ruibo Bio-Technology Co. (China) and the resulting constructs were verified by Sanger sequencing (14). Experiments were repeated three times for each.

### Reverse transcription-quantitative PCR (RT-qPCR)

Total RNA was extracted from CRC cells using TRIzol^®^ reagent (Beijing Jinbaite Biotechnology Co., Ltd., China). Reverse transcription was carried out with 2 µg RNA, which served as a template to synthesize cDNA, according to the manual of the reverse transcription kit (Zhongshi Gene Technology Co., Ltd., China). cDNA was used for detection of *CSPG4P12* mRNA using SYBR Green qPCR Mix (Zhongshi Gene Technology Co., Ltd.). GAPDH was used as an internal control gene. Primer sequences were reported in our previous study (14). The qPCR condition was 95°C for 5 min, followed by 40 cycles at 95°C for 10 s and 60°C for 20 s. The change of expression unit was calculated using the formula 2^−ΔΔCt^. Experiments were repeated three times.

### Bioinformatics analysis

This analysis used combined gene expression data from the TCGA COAD cohort (https://portal.gdc.cancer.gov/projects/TCGA-COAD) and READ cohort (https://portal.gdc.cancer.gov/projects/TCGA-READ), which contained gene expression data from 638 tumor tissues. All data were downloaded from TCGA website and analyzed using R (version: 4.2.2) to obtain *CSPG4P12* co-expressed genes. Sangerbox has a user-friendly interface and supports differential analysis, correlation analyses, pathway enrichment analysis, weighted correlation network analysis, and others (15). We uploaded the *CSPG4P12* co-expressed gene data to the Sangerbox website and used the Sangerbox website for Kyoto Encyclopedia of Genes and Genomes (KEGG) enrichment analysis. We used the GEPIA2 database (http://gepia2.cancer-pku.cn/#index) to analyze the correlation between *CSPG4P12* and common mutated genes in colorectal cancer cells.

### Cell counting kit-8 (CCK8) assay

Cell viability was evaluated by CCK8 assay. The transfected CRC cells (LOVO and Caco-2) were seeded at a density of 2×10^3^ cells/well in 96-well plates and incubated for 0, 24, 48, and 72 h. Subsequently, 10 μL CCK8 (Mei5 Biotechnology Co., Ltd., China) was added to each well and incubated for another hour at 37°C. Absorbance values at 450 nM were measured using a microplate reader (Thermo scientific, USA). Experiments were repeated three times.

### Colony formation assay

The transfected CRC cells were harvested and reseeded onto 6-well plates at a density of 2×10^3^ cells/well. After incubation for 14 days, cells were then fixed with methanol and stained with 1% crystal violet dye (0.1% w/v). Colonies were photographed (Canon, Japan) and counted by ImageJ software (NIH, USA). Experiments were repeated three times.

### Wound-healing assay

Transfected and non-transfected CRC cells (LOVO and Caco-2) were seeded onto six-well culture plates containing 2 mL RPMI 1640 medium and 10% FBS at a density of 2×10^3^ cells/well until they reached 80% confluence. The cell monolayer was scratched with a 200-μL pipette tip. The cells were then cultured in RPMI 1640 medium for 48 h. Cells and scratch closure were observed under an inverted light microscope (Nikon, Japan) and images were captured at 0, 24, and 48 h after scratching and the cell-free area was measured using ImageJ software (NIH). Experiments were repeated three times.

### Transwell assays

Transwell assays were performed to assess cell migration and invasion. For these assays, 5-10×10^4^ transfected cells in 200 μL RPMI 1640 medium were seeded onto the 8-μm pore size upper chamber (JET BIOFIL, China) with Matrigel (Corning, USA) (invasion) or without Matrigel (migration). Matrigel matrix (8-11 mg/mL) was mixed with RPMI 1640 medium at 1:4 and the final concentration of Matrigel used in transwell assays was 1.6-2.2 mg/mL. The lower chamber was supplemented with 600 μL RPMI 1640 medium containing 20% FBS. After incubation at 37°C for 24 or 48 h, cells that passed through the filter were fixed with methanol and stained with 0.1% crystal violet solution. After imaged under an inverted light microscope, cells were measured using the ImageJ software. Experiments were repeated three times.

### Western blot and antibodies

Total proteins were extracted using RIPA buffer (Beijing Zoman Biotechnology Co., Ltd, China) supplemented with the protease inhibitor cocktail (Beijing Zoman Biotechnology Co., Ltd) according to the manufacturer's instructions. Proteins (20 μg) were separated by 10% SDS-PAGE (Biotides, China) and then transferred to polyvinylidene difluoride (PVDF) membranes (Millipore, USA). The membranes were blocked in 5% milk for two hours and incubated with primary antibodies at 4°C overnight. Subsequently, the membranes were incubated with secondary antibodies at 37°C for one hour. Finally, proteins were visualized by using enzyme-linked chemiluminescence detection kit (ECL) and images were captured by a chemiluminescence imaging system (Azure Biosystems C300). The pixels from he western blot membranes were quantified by ImageJ software (NIH). Experiments were repeated three times.

NCM Universal Antibody Diluent was purchased from New Cell Molecular Biotech Co., Ltd. (China). Anti-mouse IgG antibody (H+L) (1:10000) and anti-rabbit IgG (H+L) antibody (1:10000) were purchased from Seracare (USA). Anti-beta actin (1:10000), anti-N-cadherin (1:1000), and anti-E-cadherin (1:1000) antibodies were purchased from GeneTex (USA). Anti-MMP-9 (1:1000) and anti-vimentin (1:5000) antibodies were purchased from Proteintech (China).

### Statistical analysis

IBM SPSS Statistics 26.0 software (USA) was used to perform statistical analyses. GraphPad Prism 9.0 software (USA) was used to draw graphs. A paired *t*-test was used to analyze the difference in *CSPG4P12* expression between CRC cancer tissues and adjacent normal tissues. One-way ANOVA followed by Bonferroni's *post hoc* correction was utilized to evaluate the result of wound healing assay, transwell assay, and western blot. P<0.05 was considered statistically significant.

## Result

### *CSPG4P12* expression was decreased in CRC cells and cancer tissues

**Figure 1:**
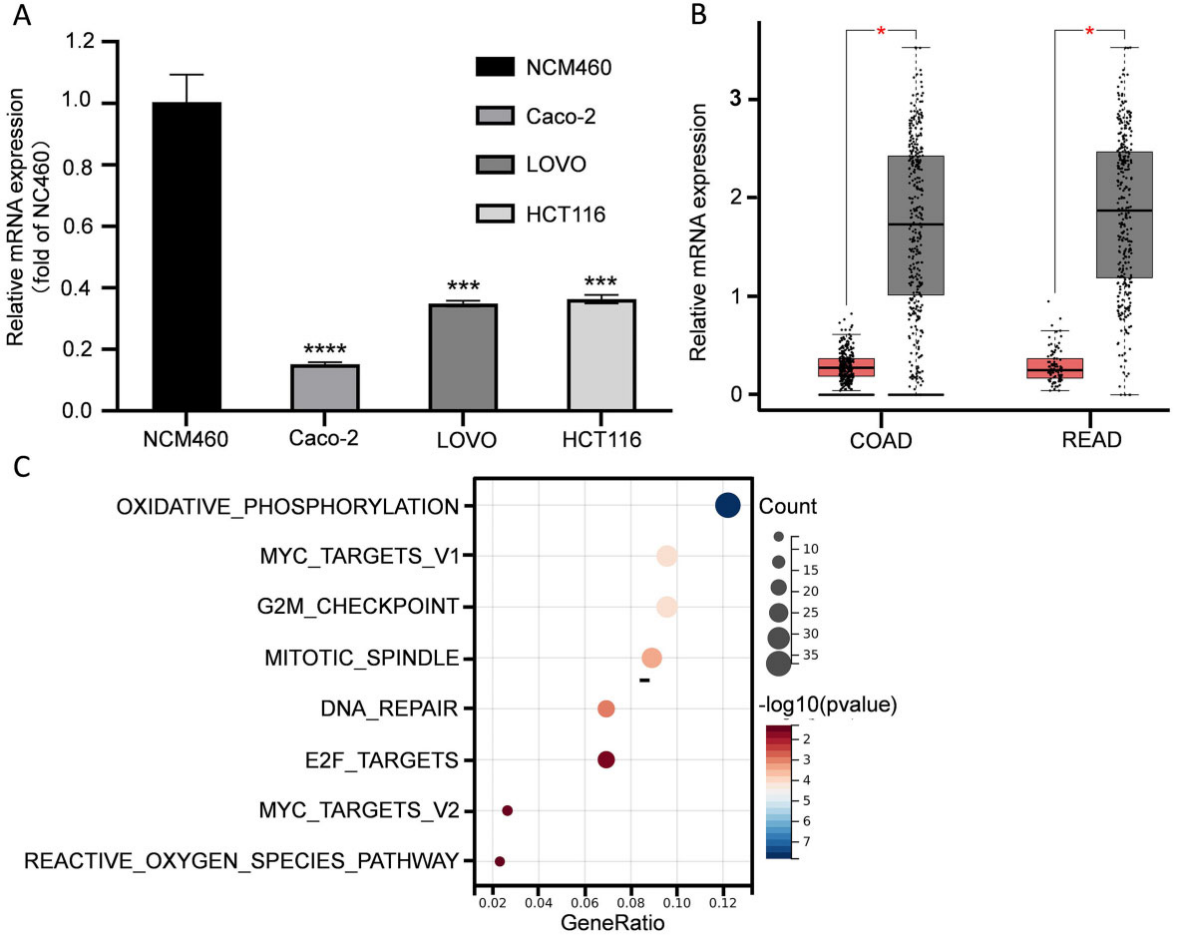
Expression of *CSPG4P12* in CRC cells and human normal colonic epithelial cell line was detected by RT-qPCR (**A**).*CSPG4P12* expression in colorectal cancer tissues (**B**) as predicted using the gene expression profiling interactive analysis database. The graph represents 275 COAD tissues (red) and 349 healthy tissues, and 92 READ tissues (red) and 318 healthy tissues. Gene expression in the combined data from the TCGA COAD cohort and READ cohort was analyzed using the CSPG4P12 co-expression screening criterion of a P-value of <0.05, and Kyoto Encyclopedia of Genes and Genomes enrichment analysis of co-expressed genes for *CSPG4P12* was performed using the Sangerbox website (**C**). Experiments were done in triplicate. Data are reported as means and SD (**A**) and median and interquartile range (**B**). ***P<0.001, ****P<0.0001 (Student's *t*-test). RT-qPCR: reverse transcription-quantitative PCR; COAD: colon adenocarcinoma; READ: rectum adenocarcinoma.

RT-qPCR revealed significantly lower levels of *CSPG4P12* expression in CRC cells. The expression level of *CSPG4P12* in Caco-2, LOVO, and HCT116 cells were 0.15-, 0.34-, and 0.36-fold compared with NCM460 cells (P<0.05; Figure 1A). The differential expression of *CSPG4P12* in CRC and normal colon-rectal tissues was analyzed by the RNA-seq data from TCGA database and GTEx database. A total of 275 colon adenocarcinoma (COAD) and 349 healthy tissues from TCGA, and 92 rectum adenocarcinoma (READ) tissues and 318 healthy tissues from GTEx were used (Figure 1B). We found that the expression of *CSPG4P12* in CRC tissues was significantly lower than that in the adjacent normal tissues (P<0.05; Figure 1B). KEGG enrichment analysis of *CSPG4P12* co-expressed genes showed that they were mainly enriched in cancer-related pathways such as G2M, MYC, and E2F (Figure 1C). Our analysis also showed no correlation between *CSPG4P12* and expression levels of commonly mutated genes in CRC patients (P53, KRAS, c-myc, Hras, Nras, Myb) (Supplementary Figure S1).

### Overexpression of *CSPG4P12* inhibited the growth of CRC cells

**Figure 2:**
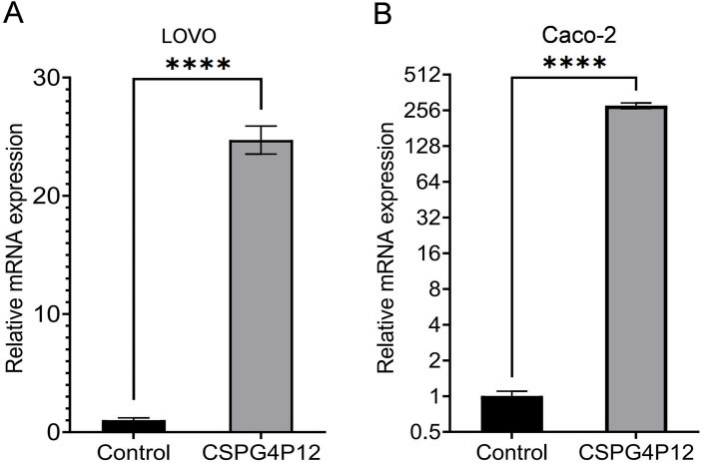
Transfection efficiency as measured using reverse transcription-quantitative PCR analysis. *CSPG4P12* expression in (**A**) LOVO and (**B**) Caco-2 cells after transfection. Data are reported as means and SD. ****P<0.0001 (Student's *t*-test). *CSPG4P12*: chondroitin sulfate proteoglycan 4 pseudogene 12. Experiments were done in triplicate.

*CSPG4P12-pUC57* overexpressed plasmid was transfected into CRC cells (LOVO and Caco-2). After 24 h, the expression levels of *CSPG4P12* in LOVO and Caco-2 cells were elevated by 24.7- and 280.2-fold, respectively (Figure 2).

**Figure 3:**
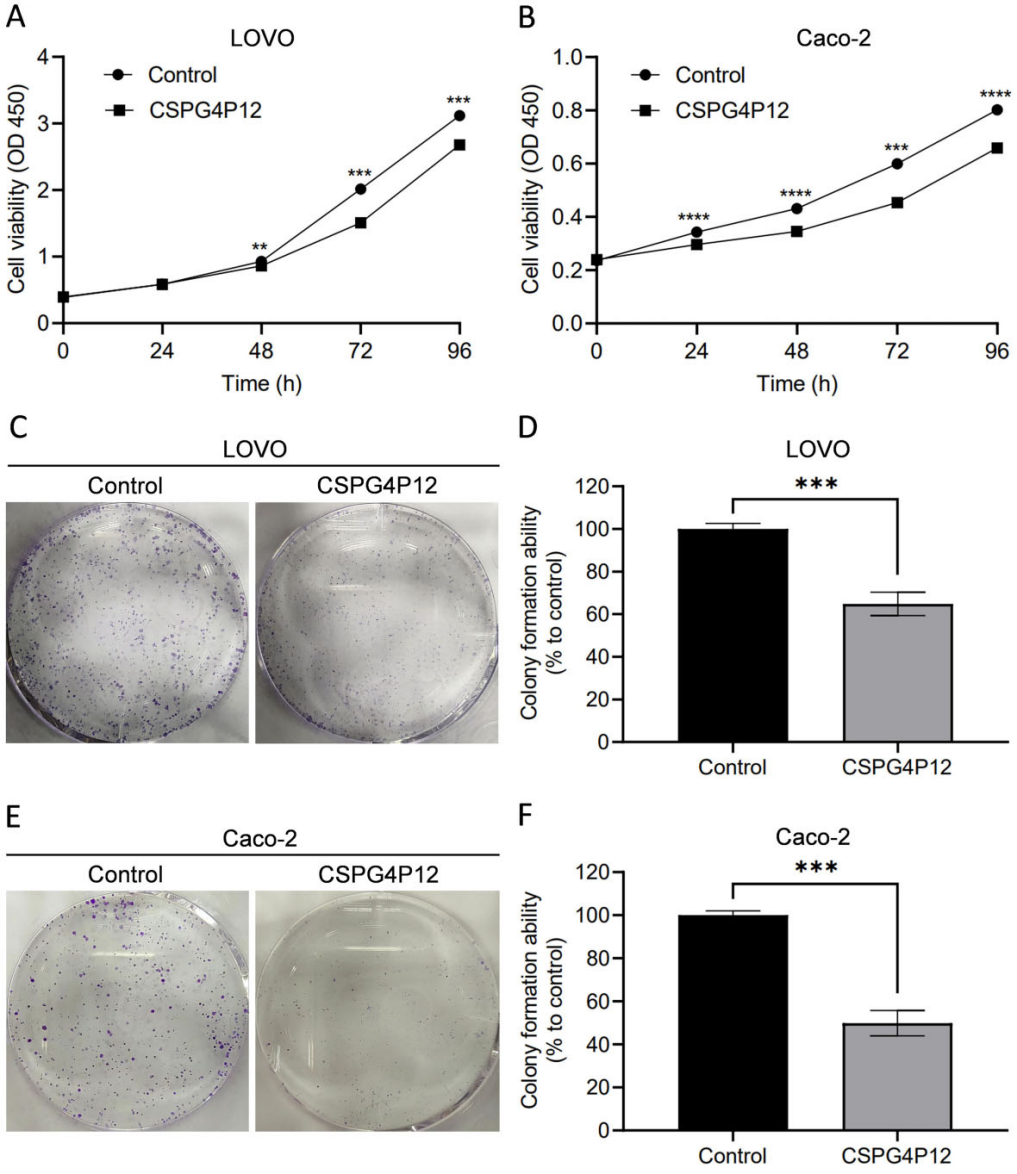
Effects of *CSPG4P12* overexpression on the proliferation of colorectal cancer cells. Detection of cell proliferation using Cell Counting kit-8 assay in (**A**) LOVO cells and (**B**) Caco-2 cells. Detection of cell proliferation ability using colony formation assay in (**C** and **D**) LOVO cells and (**E** and **F**) Caco-2 cells. Data are reported as means and SD. **P<0.01, ***P<0.001, ****P<0.0001 (Student's *t*-test). Experiments were done in triplicate.

The CCK-8 assay results showed that the number of cells decreased when LOVO and Caco-2 cells were treated with *CSPG4P12-pUC57* (P<0.01) (Figure 3A and B). The colony formation experiment also showed that the number of CRC cell colonies in the *CSPG4P12-pUC57* group was significantly lower than that in the control group (Figure 3C-F). These results demonstrated that *CSPG4P12* may promote the proliferation of CRC cells.

### Overexpression of *CSPG4P12* decreased cell migration and invasion ability

**Figure 4:**
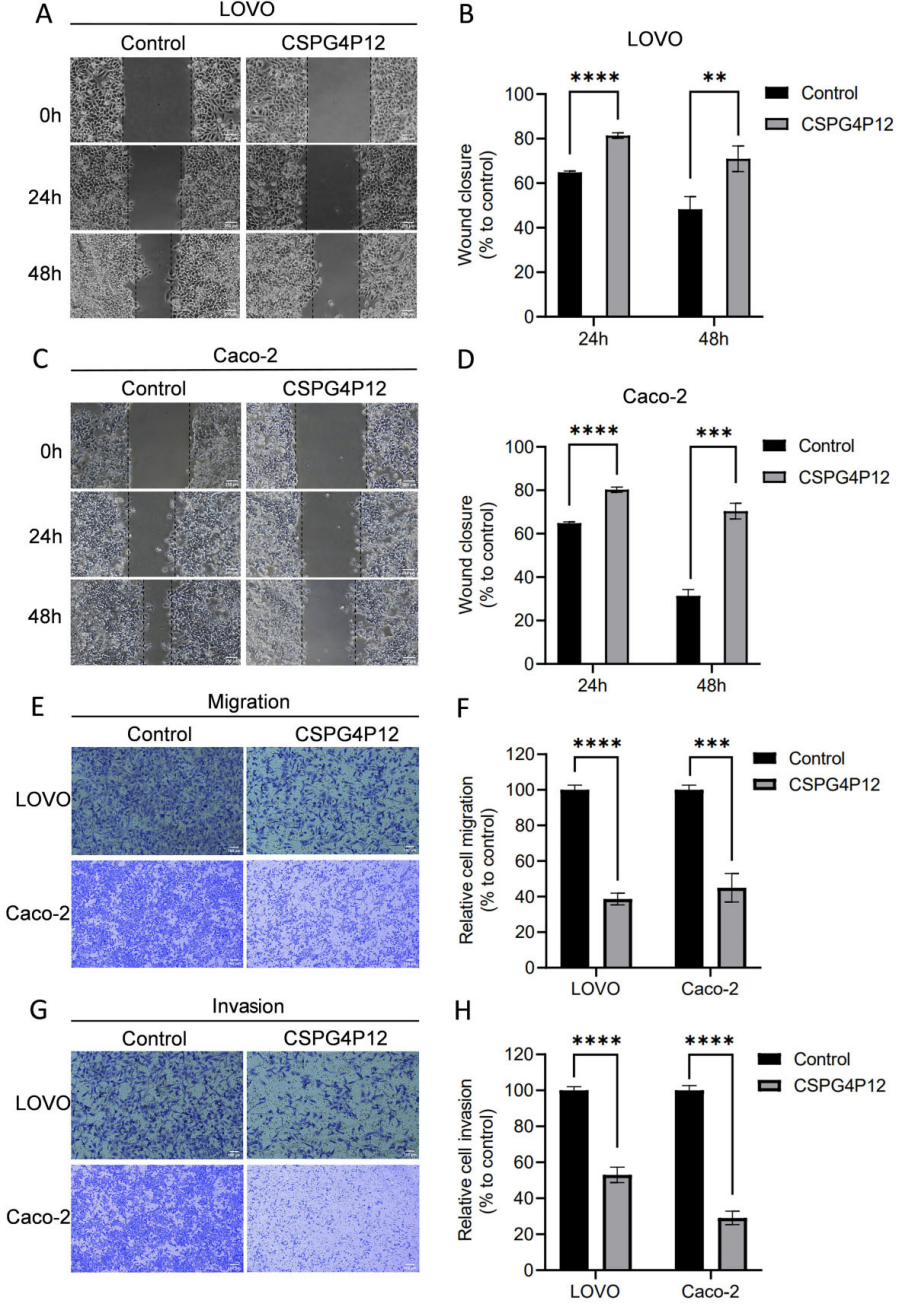
Effects of *CSPG4P12* overexpression on migratory and invasive abilities of colorectal cancer cells. Detection and quantification of cell migration using wound healing assay (magnification, ×40, scale bar 250 μM) (**A**-**D**). Detection of cell migration and invasion using Transwell assay (magnification, ×100, scale bar 100 μM) (**E** and **G**), the results of which were quantified (**F** and **H**). Data are reported as means and SD. **P<0.01, ***P<0.001, and ****P<0.0001 (Student's *t*-test). *CSPG4P12*: chondroitin sulfate proteoglycan 4 pseudogene 12. Experiments were done in triplicate.

The CRC cells with overexpression of *CSPG4P12* showed a decreased migratory ability to close the wound at 24 and 48 h (Figure 4A-D). In line with this finding, our data from transwell migration analysis demonstrated that upregulation of *CSPG4P12* repressed migration of LOVO and Caco-2 cells (Figure 4E and F). Furthermore, we observed that *CSPG4P12* overexpression suppressed cell invasive ability in LOVO and Caco-2 cells (Figure 4G and H). Our results indicated that *CSPG4P12* regulated cell migration and invasion in CRC cancer.

### Overexpression of *CSPG4P12* inhibited EMT

**Figure 5:**
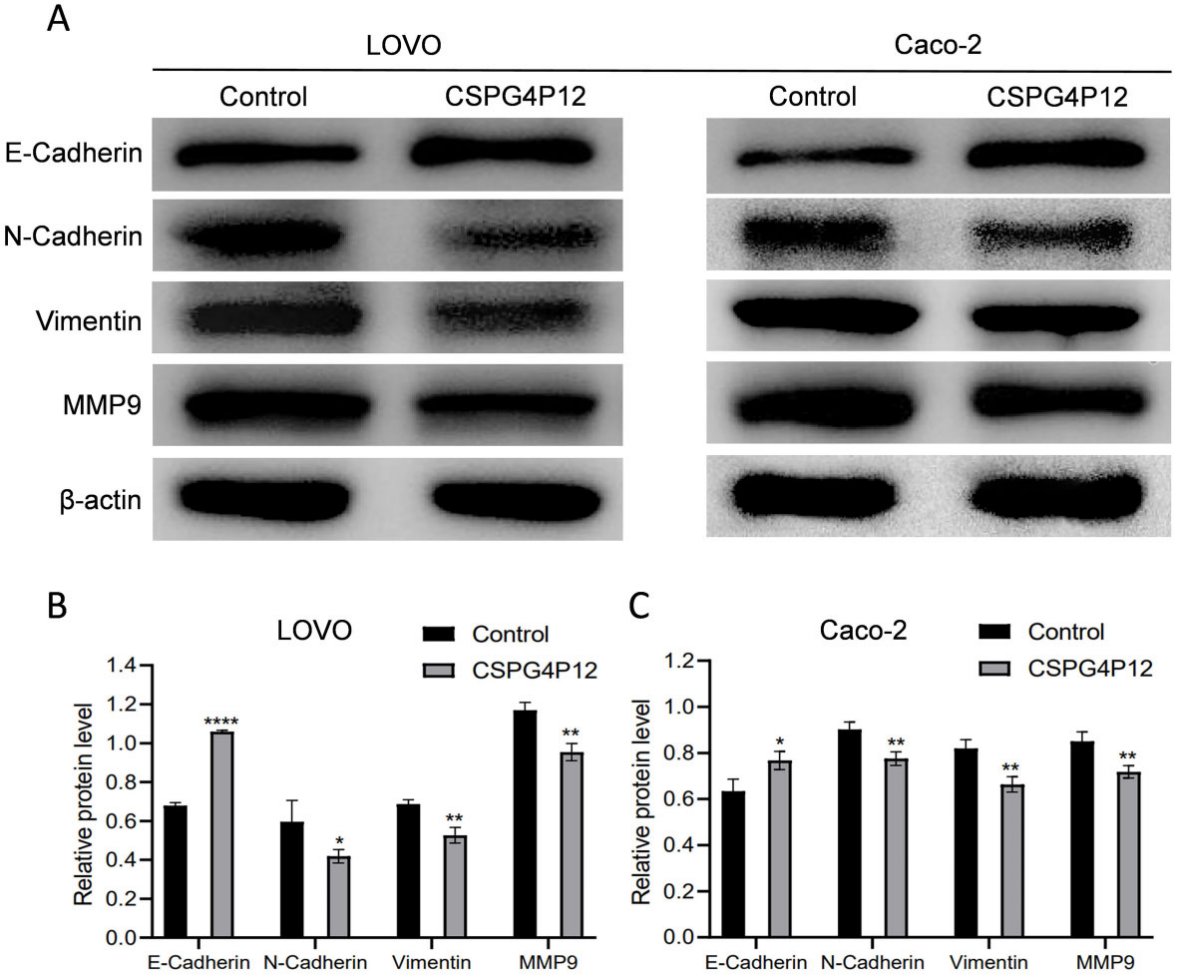
Effects of *CSPG4P12* overexpression on epithelial-mesenchymal transition marker protein expression. **A**, Detection of protein expression using western blotting, which was semi-quantified (**B** and **C**). Data are reported as means and SD. *P<0.05, **P<0.01, and ****P<0.001 compared to its own control (Student's *t*-test). *CSPG4P12*, chondroitin sulfate proteoglycan 4 pseudogene 12. Experiments were done in triplicate.

Epithelial-mesenchymal transformation (EMT) is the first step of metastasis and plays an essential role in CRC progression. Therefore, we measured the expression of EMT markers, including E-cadherin, N-cadherin, vimentin, and matrix metalloproteinase-9 (MMP9) in CRC cells with *CSPG4P12* overexpression. Western Blot assay showed that the overexpression of *CSPG4P12* inhibited the expression of vimentin, N-cadherin, and MMP9, but promoted the expression of E-cadherin, which suggested an elevated EMT progression (Figure 5A-C). These results demonstrated that overexpression of *CSPG4P12* could promote the progression of EMT in CRC.

## Discussion

Tumor metastasis is still a challenge affecting the prognosis of CRC patients. Approximately 33% of patients with CRC will develop metastases (16). Growing evidence indicates that pseudogenes, a type of long noncoding RNA (lncRNA), regulates the progression of CRC by promoting proliferation, invasion, and migration (7). For example, a study revealed that *DUXAP8* targeted miR-577 and promoted the expression of oncogene *RAB14*, which promoted colon cancer cell proliferation and progression (17).

The decreased expression of *CSPG4P12* in CRC could be attributed to several molecular mechanisms that regulate gene expression in cancer. Our analysis revealed that *CSPG4P12* co-expressed genes are significantly enriched in the E2F and MYC pathways, which are pivotal in CRC development. The E2F and MYC pathways are known to be tightly regulated under normal physiological conditions but are frequently overactivated or disrupted in cancerous cells (18,19). The enrichment of *CSPG4P12* co-expressed genes in these pathways suggested that *CSPG4P12* may act as a modulator of these critical oncogenic pathways. The downregulation of *CSPG4P12* in CRC could, therefore, be a consequence of the cancer cells' attempt to escape the normal regulatory mechanisms that limit cell proliferation and promote differentiation. By decreasing *CSPG4P12* expression, CRC cells may enhance the activity of the E2F and MYC pathways, promoting uncontrolled cell division and progression of the cancer.

*CSPG4*, which is the parental gene of *CSPG4P12* (12), is involved in tumor carcinogenesis, particularly in cancer proliferation, motility, and metastatic spread, and has been used as an independent biomarker for cancer prognosis (20). Our study disclosed that *CSPG4P12* acted as a crucial regulator in CRC development, and overexpression of *CSPG4P12* restrained CRC cell progression and metastasis. Consistent with this finding, our previous study (14) revealed that expression of *CSPG4P12* was decreased in NSCLC tissues compared to normal lung tissues, and overexpressed *CSPG4P12* significantly inhibited lung cancer cell proliferation, migration, invasion, and adhesion. CSPG targeting antibody-drug conjugate showed a strong toxic effect on cancer cells in a concentration-dependent manner (21). Furthermore, *CSPG4*-specific mAb has been shown to inhibit growth, adhesion, and migration of cancer cells *in vitro*, and significantly reduces the tumorigenic power of cancer cells and mitigates metastases and recurrence *in vivo* (22). *CSPG4* is also thought to play important roles in the progress of wound healing (23). In present work, the wound-healing assay and transwell experiment validated the stimulatory effect of *CSPG4P12* on cell migration and invasion.

EMT plays critical roles in cancer metastasis, resulting in poor prognosis in CRC patients (24). In the present study, we showed that *CSPG4P12* overexpression mediated the inhibition of EMT. Previous studies revealed that knockdown *CSPG4* can reduce expression of EMT markers (E-cadherin, N-cadherin) and key EMT regulator (Snail), and then inhibit tumor migration/invasion (25,26). In our study, western blot experiments revealed that epithelial markers (E-cadherin) were significantly increased, while mesenchymal markers (vimentin, N-cadherin) were decreased by *CSPG4P12*. A growing body of clinical and experimental studies have shown the prognostic value of these EMT‐related proteins in CRC patients. For example, studies have reported that aberrant regulation of EMT-related epithelial (E-cadherin) and mesenchymal (vimentin, N-cadherin) markers have been identified in CRC and are associated with increased rate of cancer recurrence, metastasis, and poor prognosis of CRC patients (27-30). E-cadherin is a key component of the adhesion junctions and regulates CRC proliferation and migration (31). Kuphal and Bosserhoff (32) reported that N-cadherin is directly regulated by E-cadherin, and loss of E-cadherin induces N-cadherin expression in tumorigenic EMT.

MMP9 contributes to various phases of colorectal cancer progression, including invasion, EMT, and angiogenesis (33,34). We found that overexpression of *CSPG4P12* could significantly decrease the expression of MMP9.

We acknowledge the importance of *in vivo* experiments or 3D co-culture models in bridging the gap between *in vitro* results and clinical applicability. Although resource constraints precluded us from conducting these studies at this stage, we emphasize the value such experiments would bring to the field. Specifically, future research employing *in vivo* and 3D co-culture models could further validate *CSPG4P12* as a key player in CRC progression and provide deeper insights into its mechanisms of action. These studies would not only indicate the therapeutic potential of targeting *CSPG4P12* but also facilitate the development of more effective and personalized treatment strategies for CRC patients.

In conclusion, our current study lays the groundwork for understanding *CSPG4P12*'s role in CRC. We hope that our work will inspire and pave the way for these essential investigations, moving us closer to novel interventions for CRC.

## Supplementary Material

Click here to view [pdf].
