# Supplementary material for: Pseudogene CSPG4P12 inhibits colorectal cancer progression by attenuating epithelial-mesenchymal transition
Source: Braz J Med Biol Res. 2024 May 20;57:e13645. doi: 10.1590/1414-431X2024e13645 (PMC11136487; doi:10.1590/1414-431X2024e13645)

**Figure S1.** Correlation analysis between *CSPG4P12* and expression levels of commonly mutated genes in colorectal cancer patients (KRAS, Nras, Hras, P53, c-myc, Myb).

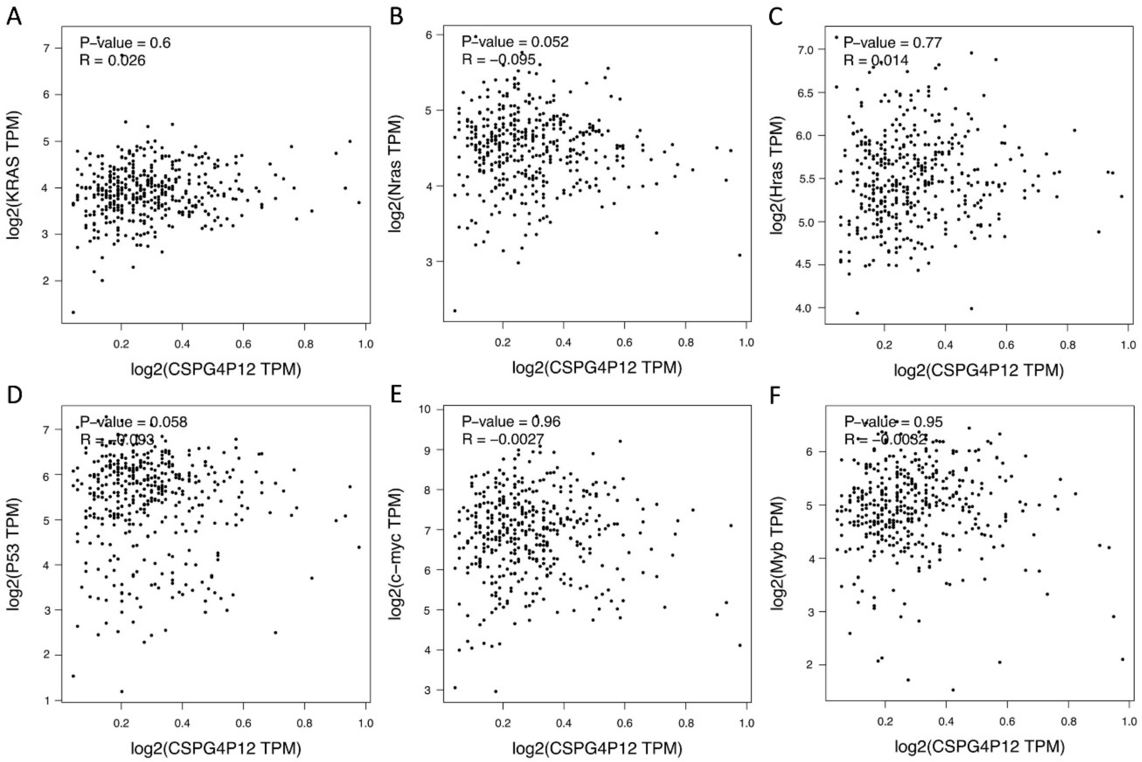

Supplement: Supplementary file 1 [file 1414-431X-bjmbr-57-e13645-suppl.pdf]
